# Are the content and usability of a new direct observation tool adequate for assessing competency in delivering person-centred care: a think-aloud study with patients and healthcare professionals in Sweden

**DOI:** 10.1136/bmjopen-2024-085198

**Published:** 2024-07-01

**Authors:** Nina Ekman, Andreas Fors, Philip Moons, Eva Boström, Charles Taft

**Affiliations:** 1 Institute of Health and Care Sciences, Sahlgrenska Academy, University of Gothenburg, Gothenburg, Sweden; 2 Gothenburg Centre for Person-Centred Care (GPCC), University of Gothenburg, Gothenburg, Sweden; 3 Department of Public Health and Primary Care, Faculty of Medicine, KU Leuven, Leuven, Belgium; 4 Region Västra Götaland, Research, Education, Development and Innovation, Primary Health Care, Gothenburg, Sweden; 5 Department of Pediatrics and Child Health, University of Cape Town, Cape Town, South Africa; 6 Department of Nursing, University of Umeå, Umeå, Sweden

**Keywords:** Quality in health care, Health Education, EDUCATION & TRAINING (see Medical Education & Training)

## Abstract

**Abstract:**

**Objective:**

To evaluate the content and usability of a new direct observation tool for assessing competency in delivering person-centred care based on the Gothenburg Centre for Person-Centred Care (gPCC) framework.

**Design:**

This is a qualitative study using think-aloud techniques and retrospective probing interviews and analyzed using deductive content analysis.

**Setting:**

Sessions were conducted remotely via Zoom with participants in their homes or offices.

**Participants:**

11 participants with lengthy experience of receiving, delivering and/or implementing gPCC were recruited using purposeful sampling and selected to represent a broad variety of stakeholders and potential end-users.

**Results:**

Participants generally considered the content of the four main domains of the tool, that is, person-centred care activities, clinician manner, clinician skills and person-centred care goals, to be comprehensive and relevant for assessing person-centred care in general and gPCC in particular. Some participants pointed to the need to expand person-centred care activities to better reflect the emphasis on eliciting patient resources/capabilities and psychosocial needs in the gPCC framework. Think-aloud analyses revealed some usability issues primarily regarding difficulties or uncertainties in understanding several words and in using the rating scale. Probing interviews indicated that these problems could be mitigated by improving written instructions regarding response options and by replacing some words. Participants generally were satisfied with the layout and structure of the tool, but some suggested enlarging font size and text spacing to improve readability.

**Conclusion:**

The tool appears to satisfactorily cover major person-centred care activities outlined in the gPCC framework. The inclusion of content concerning clinician manner and skills was seen as a relevant embellishment of the framework and as contributing to a more comprehensive assessment of clinician performance in the delivery of person-centred care. A revised version addressing observed content and usability issues will be tested for inter-rater and intra-rater reliability and for feasibility of use in healthcare education and quality improvement efforts.

**Strengths and limitations of this study**

- A strength is that nearly half of the sample used in evaluating the tool was made up of patients.
- Another strength is that the study evaluated a tool developed from an evidence-based person-centred care framework.
- A limitation is that the use of a video-recorded patient–physician interaction may have impacted the participants’ appraisals of the usability of the tool.

## Introduction

 Person-centred care is widely recognised as a key core competency needed for health professionals to provide high-quality care^1 2^ and its benefits for patients, healthcare professionals and healthcare organisations have been demonstrated in numerous studies.^2^,^11^ Signalling the importance of preparing the healthcare workforce to deliver person-centred care, several professional bodies now encourage that person-centred care be integrated in health professional education, development and training curricula,^12^,^15^ and person-centred care is now increasingly included in instructional programmes.^16 17^

Direct observation of trainees performing authentic or simulated patient care and clinical activities is an important component of health education and evaluation and is now required by several accreditation councils.^18^ Today, a multitude of direct observation tools designed to guide and structure formative and summative assessments of clinical skills are available.^19^ Numerous tools have also been developed or adapted for use in assessing competency in delivering person-centred care.^20^ As person-centred care is a complex, multidimensional construct^21^ lacking consensus regarding terminology,^22^ definitions^23 24^ and conceptual frameworks,^25^,^28^ it is unsurprising that person-centred care direct observation tools differ in their operationalisations of this concept and generally show low concurrent validity.^29^ It is noteworthy, however, that few provide readily identifiable information about the conceptual underpinnings guiding their development^20 25^ as such information is essential for understanding what the tool aims to assess and for evaluating its validity and utility in measuring person-centred care.

The purpose of this study was to evaluate a new direct observation tool for assessing person-centred care based on the framework developed by the Gothenburg University Centre for Person-Centred Care (gPCC).^30^ The gPCC framework has been widely implemented in Sweden, but also in other parts of Scandinavia, the UK and Poland,^31 32^ and empirical studies using this framework have reported, among others, improved patient self-efficacy, shortened hospital stays, increased job satisfaction among health professionals and better cost-effectiveness compared with usual care.^4^,^8^ It is also the most frequently applied framework in research on person-centred interventions^33^ and was influential in the development of the European standard for patient involvement in healthcare.^34^ Rooted in Ricoeur’s ethics of personhood and heavily influenced by Nussbaum and Sen’s capability theory approach,^35^ the gPCC framework emphasises the co-creation of care through partnerships between patients, their families and health professionals,^30 36^ and defines person-centred care as care that ‘highlights the importance of knowing the person behind the patient—as a human being with reason, will, feelings, and needs—in order to engage the person as an active partner in his/her care and treatment’.^30^ With the aim to move person-centred care research into mainstream practice, the framework proposes three core routines for practising person-centred care: *initiating the partnership* by actively listening to the patients’ narratives, *working the partnership* by mutually formulating decisions and health plans, and *safeguarding the partnership* by co-documenting and sharing health plans.^30^

Although the gPCC framework clearly outlines formal goals and tasks to be accomplished in person-centred care clinical encounters, it is less clear regarding how health professionals should be and what skills they need to effectively enact them in everyday practice. Recent interview studies with patients, relatives and health professionals conducted by the University of Gothenburg Centre for Person-Centred Care (GPCC) have contributed to a more comprehensive understanding of what behaviours, attitudes and skill sets clinicians should demonstrate when practising the framework.^37 38^ Combining the goals and tasks laid out in the gPCC framework with relational aspects derived from the above interview studies, together with knowledge gained from a review of existing person-centred care direct observation tools,^20^ we developed a new direct observation tool for assessing competency in the delivery of person-centred care. The aim of this study was to evaluate the content and usability of the tool among potential end-users and other stakeholders.

¹Although differences exist in the definitions of person-centred care and patient-centred care, the terms are frequently used interchangeably in the literature. For the sake of parsimony, the term person-centred care has systematically been used in this article.

## Method

### The direct observation tool

The tool assesses four major domains: person-centred care activities, clinician manner, clinician skills and person-centred care goals (see online supplemental appendix). The domain ‘person-centred care activities’ includes tasks and goals covering eight broad categories of activities (eg, *seeks to understand the patient’s perspective*) to be accomplished in clinician–patient interactions as per the gPCC framework. Each activity is assessed against a set of defining behavioural indicators. The domain ‘clinician manner’ includes nine attitudes or attributes (eg, *respectful*) that the clinician should demonstrate through verbal and/or non-verbal behaviours when interacting with patients in a person-centred manner. Behavioural indicators are provided for each attitude (eg, *is non-judgemental*). The ‘clinician skills’ domain describes sets of perceptual and behavioural skills needed to accomplish person-centred care goals (eg, *speaks in a manner appropriate to the patient’s level of understanding*). The domain ‘person-centred care goals’ includes general indicators that person-centred care goals of patient activation, engagement and trust are achieved (eg, *patient freely and actively voices concerns, opinions,* etc). All ratings are made on a bipolar, 4-point rating scale ranging from very unsatisfactory to very satisfactory, labelled --, -, +, ++. A response option labelled ‘doesn’t do’ is also provided for the ‘person-centred care activities’ domain for cases where the activity is not performed. Space is provided for personal notes beside each behavioural indicator (see online supplemental materials).

### Design

This is a qualitative study using think-aloud techniques with retrospective probing interviews. Think-aloud is a method where subjects talk aloud while solving a problem or performing a task.^39^,^41^ The method is therefore a unique source of information since it generates direct data on ongoing thought processes during task performance. Data were analysed using deductive content analysis.^42 43^

### Participants

Eleven patients and healthcare professionals from western Sweden with experience of receiving, working with and/or implementing person-centred care were recruited using purposeful sampling and selected to represent a variety of stakeholders and potential end-users. All participants were well acquainted with person-centred care concepts and had taken part in or lead seminars or training courses on the gPCC framework. We had no exclusion criteria, and no participants dropped out. Four of the participants were chronically ill patients who represented several national patient organisations and/or were members of various research advisory boards. Five were registered nurses (RNs) and two were physicians, all of whom were experienced in delivering and implementing the gPCC framework in clinical settings (table 1).

**Table 1:** Gender and role of interviewees

N=11	Gender	Representing
	Female	Patient representative A
	Male	Patient representative B
	Female	Patient representative C
	Female	Patient representative D
	Female	RN A
	Female	RN B
	Female	RN C
	Female	RN D
	Female	RN E
	Female	Physician A
	Male	Physician B

RNregistered nurse

### Procedure

The eligible participants were recruited from February 2022 to April 2022 through a combination of network and snowball sampling. They were first contacted by phone by the first author (NE) and subsequently a letter explaining the purpose and procedures of the study was sent to those who agreed. All the participants signed an informed consent form. The participants were given a choice to participate in person or via Zoom. All of them chose to participate via Zoom at their homes or places of work. The tool was sent to the participants a few days prior to the scheduled session and they were asked to review and familiarise themselves with it. The first author (NE) conducted all think-aloud sessions and probing interviews. The interview guideline was developed and tested within the research group as a complement to think-aloud data to cover areas not mentioned spontaneously during think-aloud sessions regarding content coverage, comprehensibility, readability, layout and response format of the tool. The guideline was pilot tested on the first participants to see if any changes were needed, which turned out not to be necessary. At the start of each session, the aim of the study and the study procedure were explained, and the participants were asked if they had any questions about the tool and if they agreed to the sessions being video-recorded via Zoom. The sessions comprised a think-aloud phase and an interview phase. In the think-aloud phase, the participants were asked think-aloud while using the tool to rate a 5-minute, video-recorded patient–physician interaction in which the patient consulted for shoulder pain in a primary care setting. In line with Ericsson and Simon’s protocols,^39^ the participants were instructed to talk aloud about their thoughts and actions, as well as any confusion or concerns they had. If the participants were silent for more than a few seconds, they were prompted to ‘please keep talking’. In such cases, they were also occasionally asked to explain what they were thinking while they were silent. They were told that the think-aloud phase would end when they had completed their ratings or otherwise wished to terminate the session.

Semistructured interviews were conducted directly after the think-aloud phase. Participants were first asked for their general impressions of the tool; thereafter, questions were asked, when necessary, regarding the content coverage, comprehensibility, readability, layout and response format of the tool (see Box 1). Questions were also asked to probe difficulties or uncertainties observed by the interviewer or verbalised by the participant during the think-aloud phase. The interviews were terminated when the interviewer judged that no new information could be gleaned or at the request of the interviewee.^39 40^ All sessions were conducted by NE and were digitally recorded and lasted 40–78 min (mean 58 min).

Box 1 **Interview guide**

- What were your general impressions of the tool?
- In general, what did you think about the content coverage of the tool?
- Were there things that you felt were missing and should be added? If so, what?
- Were there things that you felt could be omitted? If so, what?
- Were there things that you had trouble understanding? If so, what?
- Were there things that could be phrased differently? If so, what?
- What did you think about the readability of the text, for example, font size and line spacing?
- What did you think about the layout of the tool?
- Would you have preferred the tool to be organised in another way? If so, how?
- What did you think about the rating scale? Any difficulties using it?
- Would you have preferred another format for the rating scale? If so, what?
- What did you think about the written instructions? How would you change them?
- What did you think about the time it took to complete your ratings?
- Are there any other changes to the tool that you would suggest we make?

### Analysis

An explorative, qualitative deductive content analysis, inspired by Graneheim and Lundman, was used in this study.^42 43^ Reflecting the over-riding aims of the study, the main categories in the analyses were defined as content coverage and usability issues. First, three of the authors (NE, AF and CT) independently viewed the think-aloud and interview recordings. NE both transcribed the interviews and read through the transcribed interview texts several times to obtain a sense of the whole. Subsequently, meaning units comprising words, sentences or phrases corresponding to the defined main categories were identified. In the next step, meaning units were condensed, coded and sorted into subcategories for each of the main categories. To improve the trustworthiness of the analyses, at least two of the authors (CT, AF) collaborated with the first author (NE) regularly in the analysis and discussed their results until a consensus was obtained. No coding software was used.

### Patient and public involvement

The study was conducted within the GPCC, where patient and public involvement is essential. Several patient representatives were involved in the present study.

## Results

The two predetermined main categories, content coverage and usability issues, along with their respective subcategories and participants’ suggestions for improving the instrument, are shown in table 2.

**Table 2:** Categories, subcategories and participant suggestions for change

Categories	Subcategories	Participant suggestions
Content coverage	Comprehensiveness/relevanceRedundancy	Expand: activity ‘resources and capabilities’Expand: activity ‘patient’s psychosocial needs’Add: checks how patient perceives diagnosis and treatment informationCorrect: overlap between domains manner and skillsReduce: number of example behaviours given
Usability issues	ComprehensionReadabilityLayoutRating scale formatGeneral usability	Replace: uncommon words, for example, mutual gazeIncrease: font size and line spacingChange: one page per domainChange: 4-point to 5-point scaleAdd: labels for anchorsDefine: ‘doesn’t do’ optionAdd: more explicit instructions

### Content coverage

The category ‘content coverage’ comprised the subcategories ‘comprehensiveness/relevance’ and ‘redundancy’.

#### Comprehensiveness/relevance

The participants commented on the content mainly during the follow-up interviews, either spontaneously or after prompting. Overall, they reported that the content of the instrument was comprehensive and relevant for assessing person-centred care in general and gPCC in particular. However, some participants pointed to the need to include a wider array of indicators, particularly related to patients’ own resources, to cover the activities ‘attends to patient’s psychosocial needs’ and ‘identifies and supports patient’s personal capabilities’ to better reflect the emphasis on these activities in the gPCC framework.

Capabilities and resources in the patient must be more visible in the instrument because awareness of it is unusual in good care compared to person-centred care. (RN B)

Some participants also commented that although the gPCC framework defines what the clinician should do and try to achieve in their interactions with patients, it focuses less on how clinicians should be with the patient and what skills they need to successfully achieve person-centred care goals. They therefore felt that the addition of the domains ‘clinician manner’, ‘clinician skills’ and ‘goals’, as well as the activities ‘making a personal connection’ and ‘co-sets agenda’, contributes to more comprehensive assessments of person-centred care.

Some participants commented that when communicating medical information, the clinician should be attentive and responsive to how the patient experiences and understands it; therefore, they suggested that the activity ‘attends to patient’s information needs’ should be expanded to cover this. Another participant suggested that as building partnerships with patients is a central goal of person-centred care, an item evaluating how well partnership was achieved should be added to the ‘goal’ domain.

It is sometimes quite hard to achieve partnership and it should be a clear part of the instrument because it is what person-centred care is all about. (RN D)

#### Redundancy

Some participants explained that the domains ‘clinician manner’ and ‘clinician skills’ appeared to overlap in that the behavioural and perceptual skills included in the tool encompass some of the attitudes/attributes included in clinician manner.

I had a problem in seeing the difference between manner and skills. (RN A)

When rating clinician manner, some of the participants interpreted the example behavioural indicators, meant to illustrate each attitude/attribute, as a checklist of behaviours, where ratings were based on the number of example behaviours the clinician exhibited.

The physician shows some of the verbal behaviors listed here but not so many of the non-verbal ones, so I’ll rate her lower. (Patient C)

### Usability issues

Analyses of the interview and think-aloud material pointed to several usability issues related mainly to item wording, layout, instructions and response options. The category ‘usability issues’ included the subcategories ‘comprehension’, ‘readability’, ‘layout’, ‘rating scale format’ and ‘general usability’.

#### Comprehension

The participants reported that the language used in the instrument was generally easy to understand, but some were not familiar with a few expressions, for example, ‘go the extra mile’ and ‘mutual gaze’. The subheading *paraverbal behaviours* was not understood by most participants.

I don’t understand what ‘mutual gaze’ means. Is that a well-known expression? (Patient A)

#### Readability

Most of the participants were satisfied with the font size and line spacing. One stated that he/she would have preferred larger text and line spacing in the printed version.

When I printed out the instrument on paper it was bit compact, with small text, but I can enlarge the letters when it is on the screen. (Patient D)

#### Layout

Some participants felt the instrument was too compact and suggested that spreading it out over more pages, with one domain per page, would facilitate its use.

#### Rating scale format

Issues regarding the rating scale mainly concerned whether a 4 or 5-point rating scale should be used, if the scale needed verbal anchors, as well as how the ‘doesn’t do’ response option was used. There was no consensus among the participants on any of these issues. Some thought the 4-point, plus–minus rating scale was unambiguous, while others felt that a midpoint and verbal anchors would be preferable; however, no one felt that the format influenced their ratings appreciably. On the other hand, some of the participants were observed to use the ‘doesn’t do’ option as the lower endpoint of the rating scale, rather than as a means to indicate that the activity did not occur.

It would be easier to rate if there was a middle point on the rating scale. (RN E)

It’s easier with plus and minus in the rating scale, you know what they mean*.* (Patient A)

#### General usability

Most of the participants thought the tool was easy to use and felt that it would be useful for assessing person-centred care in educational and clinical settings.

It is easy to follow, you just start at the beginning and work right through it*.* (Physician B)

However, several participants wanted the interviewer to walk them through the tool at the beginning of the session to ensure that they had correctly understood the written instructions.

Could you please go through the instrument before we start, just to make sure I have understood it right*.* (Patient B)

## Discussion

Although the gPCC framework has been applied and tested in numerous clinical studies and implemented in clinical practice in several countries, no direct observation tool built upon this evidence-based framework exists to guide and structure formative and summative assessments in professional training and education. This article presented a newly developed direct observation tool for this purpose and evaluated its content and usability for assessing competency in the delivery of person-centred care.

In keeping with common conceptions of competency, this tool was developed with the aim to assess knowledge, skills and attitudes, that is, knowing what to do, how to do it and how to be, to achieve person-centred care goals. The gPCC framework with its three routines of initiating, working and safeguarding partnerships served as a blueprint for defining the tasks and goals to be accomplished in person-centred care patient encounters (ie, what to do). Although the gPCC framework defines tasks and goals to be accomplished in person-centred care patient encounters, it gives little guidance as to how clinicians should actually be with patients. The participants were generally satisfied with the tool’s coverage of these elements; however, in order to discriminate between good care and person-centred care, they suggested that the tool should place greater emphasis on eliciting and addressing both patients’ capabilities and resources as well as their psychosocial needs to better reflect their importance in the gPCC framework. A defining feature in the framework is to build a partnership, which was also suggested to be more prominent in the instrument, both by promoting patients’ self-efficacy by identifying and using their personal capabilities, as well as by supporting their psychosocial needs. Identifying and addressing patients’ resources and needs require health professionals to be aware of the interpersonal nature of clinical encounters and that they can be strengthened or ignored in those interactions.^44 45^ In this context, an important dimension is that patients’ suffering is sometimes reduced to physical or mental pain alone. However, Ricoeur^35^ suggests that the most challenging suffering occurs when human capabilities are not acknowledged, relegating individuals to less than their full potential.^35^

The distinction between ‘doing person-centred care’ and ‘being person centred’ has been discussed in earlier studies.^37 46^ As shown in these studies, being person centred involves interacting with the patient in an empathic, compassionate, courteous, respectful, interested and genuine manner, as well as demonstrating behavioural and perceptual skills such as active listening and interpreting patients’ non-verbal emotional cues. Wolf *et al* found in an interview study that professionals tend to focus on tasks and goals to be achieved, whereas patients are concerned with how the clinician acts towards them.^46^ The concepts ‘presence’ and ‘attentiveness’ have been used to describe how responding to cues that maximise resources in daily life can be performed.^47 48^ One of the core aspects in person-centred practice has been described by McCormack and McCance as being in relation and being in place which emphasises the importance of relationships and to be interconnected with one’s social world.^48^

Few usability issues were observed during the think-aloud sessions or mentioned by the participants during subsequent probing interviews. In general, the participants needed no further guidance for using the tool than that provided in the instructions, which suggests that the tool was intuitive and easy to use. On the other hand, some of the participants found it difficult to understand the ‘doesn’t do’ alternative, intended for designating activities that did not appear in the interactions, and so we have clarified its use in the instructions. Also, some words such as ’paraverbal’ and ‘mutual gaze’ were not understood by all participants and were therefore replaced with more common words in line with recommendations.^49^

Furthermore, the participants expressed a slight preference for a 5-point Likert scale with a neutral midpoint to our 4-point scale. The potential effects of a 4-point vs 5-point scale on observer ratings have been extensively debated and investigated for decades; however, structural differences, including number of scale points and labelling, seem to have only marginal impact on rating behaviour or in decreasing rater variance.^50 51^ As other studies have shown, a rater preference for a 5-point rating scale,^52^ we have opted to change to a 5-point scale in the revised version of the tool.

One of the main challenges to implementing person-centred care is that clinicians often feel they are already practising it.^8^ Direct observation of trainees while performing clinical activities, coupled with timely, detailed and actionable developmental feedback, is an effective means to help them gain insight into their strengths and weaknesses.^53^ This tool may be of use in guiding and structuring both what should be observed in patient encounters and what feedback is needed for formative development. Further evaluation of the tool is necessary for high-stakes use.

### Strengths and limitations

A strength in the present direct observation tool is that patients represented almost half of the participants in both development studies. Although patient involvement in the development of person-centred care assessment instruments has long been emphasised,^54^ none of the existing person-centred care direct observation tools were developed with patients or their families.^20^ Another strength is that we used an evidence-based person-centred care framework in the development of the tool, whereas few other tools are transparent as to which, if any, conceptual framework they are based.

There are several limitations to this study. The use of a video-recorded patient–physician interaction may have impacted the participants’ positive appraisals of the usability of the tool. Since participants were allowed to stop, replay and return to previous video sequences when performing their ratings, the cognitive burden normally associated with observational assessments of real-life interactions may have been lessened. Hence, our usability results may not be generalisable to other observation formats. Another limitation is that we cannot make any claims of reaching saturation in any of these categories; the richness of the data suggests on the other hand that we came a long way and that the number of participants chosen was adequate. We judged that saturation had been approached in the data because later probing interviews added little new information to what earlier probing interviews had already provided.

Due primarily to social distancing during the COVID-19 pandemic, the sessions were conducted remotely via the video-conferencing platform Zoom rather than in person. Although such platforms offer advantages regarding flexibility, efficiency, convenience and cost-effectiveness, there is little research on their effects as a data collection method in qualitative research.^55^ Earlier studies using less advanced platforms (eg, Skype) have suggested difficulties in developing rapport in online interviews, partly owing to technical problems with the platforms; however, recent studies using Zoom with interviews^55 56^ and think-aloud found it to be easy to use and yield valid results.^57^

## Conclusion

The direct observation tool appears to satisfactorily cover major person-centred care activities outlined in the gPCC framework. The inclusion of content concerning clinician manner and skills was seen to contribute to more comprehensive evaluations of clinician competency in the delivery of person-centred care. A revised version addressing observed content and usability issues will be tested for inter-rater and intra-rater reliability and for feasibility of use in healthcare education and quality improvement efforts.

## supplementary material

10.1136/bmjopen-2024-085198 online supplemental file 1

## Data Availability

All data relevant to the study are included in the article or uploaded as supplemental information.
