# Supplementary material for: Are the content and usability of a new direct observation tool adequate for assessing competency in delivering person-centred care: a think-aloud study with patients and healthcare professionals in Sweden
Source: BMJ Open. 2024 Jul 1;14(6):e085198. doi: 10.1136/bmjopen-2024-085198 (PMC11328633; doi:10.1136/bmjopen-2024-085198)
Supplement: online supplemental file 1 [file bmjopen-14-6-s001.pdf]

Supplementary materials: The tool (preliminary version)

**Initiating the partnership**							
**Activities**	**Behavioral indicators**	**Doesn't do**	**- -**	**-**	**+**	**++**	**Comment**
Makes a personal connection	Greets patient in a warm and respectful manner						
	Attempts to make patient feel at ease						
	Is responsive to patient's emotional and physical state						

Co-sets agenda	Seeks to elicit all patient's aims and reasons for meeting						
	Discusses and prioritizes agenda items.						

Seeks to understand patient's perspective	Actively and attentively listens to patient's concerns						
	" feelings						
	" illness beliefs						
	" illness experiences						
	" psychosocial impacts of illness						
	" treatment goals						

	Verifies that he/she understands patient's viewpoints correctly						
	Validates patient's perspective						

**Working the partnership**							
Attends to patient's information needs	Gives information appropriate and relevant to the patient's needs and wants						
	Gives information in a manner that the patient can understand						
	Clarifies own reasoning and uncertainty						

Finds common ground/ engages in shared decision-making	Identifies and synthesizes areas of agreement and disagreement						
	Seeks to reconcile and align patient-clinician understanding and goals						
	Proposes and discusses care and treatment options compatible with patient's wants						
	Solicits patient's suggestions, opinions, preferences						

Attends to patient's psychosocial needs	Identifies and discusses emotional and social impacts and support needs						

Identifies and supports patient’s personal capabilities	Helps patient discover personal capabilities and resources for coping with illness						
	Discusses how capabilities and resources may be utilized						

**Safeguarding the partnership**							
Co-plans and documents care	Co-plans and co-documents steps to achieve and monitor shared goals						
	Solicits and encourages patient’s contribution						
	Discusses roles and division of responsibilities						
	Writes journal entries in language patient understands						
	Checks if patient perspectives are fully documented						
	Seeks final approval of documentation						
	Ensures patient access to documentation						

**Manner**	**Behavioral indicators**	**- -**	**-**	**+**	**++**	**Comments**
Courteous	*Verbal*: Introduces him/herself; addresses patient by name<br>*Nonverbal*: Makes eye contact; knocks before entering room					

Attentive and interested	*Verbal*: Shows interest in patient by e.g., “I’m curious about…”, “Please tell me more about…”					

	Nonverbal: Maintains appropriate eye contact; forward lean; open posture; body oriented toward patient					
Unhurried	*Verbal*: Engages in small talk<br>*Nonverbal:* Sits; respects pauses in conversation					
Caring/ empathic	*Verbal*: Responds to emotional cues e.g., ”That must be … frightening, disturbing, painful…”; shows concern for patient’s physical and mental comfort, e.g., “Are you comfortable in that chair”<br>*Nonverbal*: Touch; smile; proximity; nods*; Paraverbal:* soft voice tone					
Encouraging	*Verbal*: Uses open-ended questions; uses phrases, e.g., “Please tell me (more) about…”, “Go on”<br>*Nonverbal*: nods; gestures					
Respectful	*Verbal*: Is non-judgmental (suspends judgment), positive and affirming<br>*Nonverbal*: Doesn´t interrupt; shows turn-taking					

Genuine	*Paraverbal/ nonverbal:* Uses voice tone, gestures, and facial expressions congruent with verbal message					
Altruistic/ Committed	*Verbal:* shows willingness to do more than required, Go the extra mile, sees work as more than a job; shows no self-interest					
Forthright/ honest	*Verbal*: Admits uncertainty; explains own negative behavior, e.g., why stressed, irritated, etc.					

**Skills**	**Behavioral indicators**	**- -**	**-**	**+**	**++**	**Comments**
Perceptual	Carefully monitors and interprets patient's nonverbal emotional cues					
Behavioral	Speaks in a manner appropriate to the patient's level of understanding					
	Avoids jargon					
	Uses active, reflective and empathic listening skills					
	Effectively paraphrases and summarizes					

**Goals**	**Behavioral indicators**	**- -**	**-**	+	++	**Comments**
Patient activation	Gives opportunities and encourages patient to talk (clinician doesn't dominate conversation)					
Patient trust/ engagement	Free flow of conversation (turn-taking)					
	Patient freely and actively voices concerns, expectations, beliefs, opinions, suggestions, preferences					
	Mutual gaze					
