# Supplementary material for: Are the content and usability of a new direct observation tool adequate for assessing competency in delivering person-centred care: a think-aloud study with patients and healthcare professionals in Sweden
Source: BMJ Open. 2024 Jul 1;14(6):e085198. doi: 10.1136/bmjopen-2024-085198 (PMC11328633; doi:10.1136/bmjopen-2024-085198)
Supplement: Supplementary file 2 [file bmjopen-2024-085198.reviewer_comments.pdf]

PEER REVIEW HISTORY

BMJ Open publishes all reviews undertaken for accepted manuscripts. Reviewers are asked to complete a checklist review form (**http://bmjopen.bmj.com/site/about/resources/checklist.pdf**) and are provided with free text boxes to elaborate on their assessment. These free text comments are reproduced below.

ARTICLE DETAILS

**TITLE (PROVISIONAL)**	Is the content and usability of a new direct observation tool adequate for assessing competency in delivering person-centered care: a think-aloud study with patients and healthcare professionals in Sweden.
**AUTHORS**	Ekman, Nina; Fors, Andreas; Moons, Philip; Boström, Eva; Taft, Charles

VERSION 1 – REVIEW

**REVIEWER**	Frerichs, Wiebke<br>University Medical Center Hamburg-Eppendorf, Department of Medical Psychology
**REVIEW RETURNED**	20-Mar-2024

**GENERAL COMMENTS**	Dear authors,<br><br>Thank you for the invitation to review the manuscript entitled: A new direct observation tool for assessing person-centred care: Evaluation of content and usability using think aloud and probing techniques. The manuscript represents a comprehen-sive approach to the development of a new observational tool.<br>It is well written and possible for me to follow. However, I do have some feedback regarding your structure/wording as well as result section.<br>Wording:<br>- There are some wordings I find difficult to follow and advice you to check the complete manuscript again for the use of some wordings/sentences. Examples are page 5, ll 27-28 "…or adapted for use specifically in assessing competency in delivering PCC; Page 5 ll 32 unsurprising vs. not surprising; page 5 ll 49 "and empirical studies using this framework …" however, you only refer to one as an example, so possibly only say "and an empirical study using this frame-work…"<br>- Terminology regarding assessing competency or competencies or competence? Please check again where which is useful as I have the impression these two terms are mixed within the manuscript.<br>- Terminology regarding PCC but you use literature for definition of patient centered care…. possibly also state earlier in the introduction that there is a mixed use of patient vs person centered care and that you use PCC here as an ab-breviation for person centered care, however you are aware that it is also used for

	patient centered care? It just gets me early on in the first sentence, as for “my” research terminology PCC is patient-centered care.<br>- Page 6, ll 26 “conducted by the Centre” --> which centre? Please clarify<br>- Page 7, ll 50 “The informants” –> please change to eligible participants<br>Methods:<br>- I am missing where the participants have been recruited (Country, which type of clinical networks etc.) I assume in Sweden? Also, were there participants who wanted to join but were not eligible? And if so, can you be more precise about the in and exclusion criteria of these 11 participants?<br>- I am still wondering if you pre-tested the interview guideline, or how /why it was developed in the way it was used? Also, was the person interviewing the participants experienced in this? What was/is their background?<br>- Why did you choose the analysis by Graneheim and Lundman? Please provide context/reasoning here. Also, which software did you use? Where the interviews transcribed?<br>-<br>Results:<br>- I am missing a socio demographic overview of the participants: how many were female, what age groups, etc. Please provide that context at the beginning of the result section<br>- Also, you jump right into the categories and are quickly deep into the results itself. I always prefer a bit of context here, how long were the interviews, how did the subcategories evolve etc. Please provide a bit more “background” in-formation<br>- If I have that information I am able to go into the results in more detail and check again content wise and if clear to me.<br>Discussion:<br>I am missing a kind of point what type of changes you are going to do now after this testing and what this entails for the new updated version you want to test psychometrically. Please provide more in depth details here, a kind of implication section.<br>Reference:<br>- Please provide the English translation for the Swedish reference #23<br>Thank you again and I am looking forward reading your revision

**REVIEWER**	Byrne, Amy-Louise<br>Central Queensland University School of Nursing and Midwifery
**REVIEW RETURNED**	09-Apr-2024

**GENERAL COMMENTS**	Well done, another strong body of work coming from Sweden. I do enjoy reviewing this work.<br>Overall, a strong article. A few points to consider.<br>1. Please change to article rather than paper<br>2. I am a little concerned about the recruitment method. Using a network from one researcher is not an overly rigorous method or approach. I wonder if you might better explain this as a snowball sample or other more academic language?<br>3. Interesting use of technology and think aloud- strong work here<br>4. What you have done here is a review of person-centred practice. I know the liteature tends to amalgamate PCC and PCP as one and the same, but I see them as two separate (and equally

	important) elements. Person-centred care is the structures and environment around which centredness can exist (you alluded to accreditation etc in the background). PCP on the other hand is the practice elements that professionals bring. Please consider how separating these terms, or at least giving some nod to the difference in this paper. I have written on this topic myself but is still under review (I appreciate that is of little help to you now), but something I think needs to be considered in this space moving forward.

**REVIEWER**	Holmstrom, Inger<br>Maladalen University Sweden, School of Health, Care and Social Welfare
**REVIEW RETURNED**	15-Apr-2024

**GENERAL COMMENTS**	Thanks for giving me the opportunity to read and comment on this interesting paper. This small-scale qualitative study set out evaluate the content and usability of a new direct observation tool for assessing competency in delivering person-centred care based on the Gothenburg Centre for Person-Centred Care framework, by using think-aloud techniques with 11 informants, both patients and professionals. The manuscript is topical and should be interesting and relevant for researchers, clinicians and educators. A strength of the study is the integration of PCC theories and clinical work. The manuscript fits well into the aims and scope of BMJ Open. It has a good structure, is well written and easy to read and follow. I have some comments, suggestions and questions which are elaborated below.<br>Title, abstract and key words<br>The title and key words are well chosen, and the abstract summarizes the manuscript in a good way.<br>Introduction and literature-review<br>The introduction and literature-reviews are well written, cover several important points and provides a good overview of the area. The authors use a number of references referring to patient-centred care, a concept which indeed has many similarities with person-centre care, but also differences. Please see if some of theses references could be exchanged to fit better with the person-centred approach of the manuscript.<br>The study rationale is clearly described.<br>Methods<br>The data collection includes a lot of relevant information, but there are some unanswered questions:<br>Why were this number of informants chosen? Do you consider data to be saturated (or if you dislike this GT-concept, you could use “information power” a concept launched by Malterud et al 2016)?<br>I would like some more information about the participants, like age, gender, years in the profession (if applicable) etc. This demographic information could be displayed in a table.<br>Why use convenience sampling?<br>The description of the data analysis should be more comprehensive, to enhance trustworthiness. The steps in the analysis could be displayed in a table or figure, with examples of coding and categorisation.<br>Findings

	The finding are well described, and the section is easy to read and follow. The quotes are illustrative. I would however have liked if you differentiated between the informants, like RN A, Patient B etc. This to enhance confirmability, that the findings are based on contributions from all informants and not just a few.<br>Discussion and conclusions<br>The discussion is well written and interesting.<br>The study strength and weaknesses should be addressed. Preferably in relation to criteria for quality assessments of qualitative studies. You could use the CORTEQ guidelines or Lincoln & Guba's quality criteria for qualitative studies. One limitation is the use of convenience sampling. A strength is the inclusion of both patients and professionals.

VERSION 1 – AUTHOR RESPONSE

**Reviewer #1**	**Remarks:**
Thank you for the invitation to review the manuscript entitled: A new direct observation tool for assessing person-centred care: Evaluation of content and usability using think aloud and probing techniques. The manuscript represents a comprehensive approach to the development of a new observational tool.<br>It is well written and possible for me to follow.	Thank you very much for this nice comment!
There are some wordings I find difficult to follow and advice you to check the complete manuscript again for the use of some wordings/sentences. Examples are page 5, ll 27-28 "…or adapted for use specifically in assessing competency in delivering PCC; Page 5 ll 32 unsurprising vs. not surprising; page 5 ll 49 "and empirical studies using this framework …" however, you only refer to one as an example, so possibly only say "and an empirical study using this frame-work…"	Thank you for raising this issue. We have deleted "specifically" from the first sentence in hope that it makes the sentence more understandable.<br><br>"Unsurprising" and "not surprising" are to our understanding synonymous; however, if you think it will make the sentence more understandable we will naturally substitute.<br><br>Thank you, we have changed to a reference which is an overview of several conducted empirical studies.
Terminology regarding assessing competency or competencies or competence? Please check again where which is useful as I have the impression these two terms are mixed within the manuscript.	Thanks for pointing this out. We have changed all instances of "competence/ competences" to "competency/ competencies" throughout.
Terminology regarding PCC but you use literature for definition of patient centered care…. possibly also state earlier in the introduction that there is a mixed use of patient vs person centered care and that you use PCC here as an abbreviation for	Thank you for raising this issue, we have now removed the abbreviation PCC and written person-centred care throughout the manuscript. We have also added a footnote explaining our use of person-centred care.

person centered care, however you are aware that it is also used for patient centered care? It just gets me early on in the first sentence, as for "my" research terminology PCC is patient-centered care.	person-centred care<br><br>[1] Although differences exist in the definitions of person-centred care and patient-centred care, the terms are frequently used interchangeably in the literature. For the sake of parsimony, the term person-centred care has systematically been used in this article.
Page 6, ll 26 "conducted by the Centre" --> which centre? Please clarify	Thank you for making us aware of this. We have clarified that now in the manuscript "conducted by the University of Gothenburg Centre for Person-Centred Care".
Page 7, ll 50 "The informants" –> please change to eligible participants	We have now changed "the informants" to eligible participants.<br><br>The eligible participants
I am missing where the participants have been recruited (Country, which type of clinical networks etc.) I assume in Sweden? Also, were there participants who wanted to join but were not eligible? And if so, can you be more precise about the in and exclusion criteria of these 11 participants?	Thank you for noticing this. We have now clarified that the patients and healthcare professionals participating in this study were recruited in western Sweden and that the inclusion criteria were that participants with experience of receiving, working with and/or implementing person-centred care. They were ecruited using purposeful sampling and selected to represent a variety of stakeholders and potential end-users. All participants were well acquainted with person-centred care concepts and had taken part in or lead seminars or training courses on the gPCC framework. We had no exclusion criteria.<br>Eleven patients and healthcare professionals from western Sweden with experience of receiving, working with and/or implementing person-centred care were recruited using purposeful sampling and selected to represent a variety of stakeholders and potential end-users. All participants were well acquainted with person-centred care concepts and had taken part in or lead seminars or training courses on the gPCC framework. We had no exclusion criteria, and no participants dropped out.
I am still wondering if you pre-tested the interview guideline, or how /why it was developed in the	The guideline was developed and used to complement think-aloud data. Questions from

way it was used? Also, was the person interviewing the participants experienced in this? What was/is their background?	the guideline were asked only when participants did not comment on content coverage, comprehensibility, readability, layout or response format of the tool. The guideline was discussed and tested within the research group, and pilot-tested with the first two participants (no changes were needed). The first author (RN, MSc, PhD student) interviewed all participants. She has experience in conducting interviews with patients and healthcare professionals in a number of earlier studies We have now added this to the manuscript.<br><br>The first author (NE) conducted all think-aloud sessions and probing interviews. The interview guideline was developed and tested within the research group as a complement to think-aloud data to cover areas not mentioned spontaneously during think-aloud sessions regarding content coverage, comprehensibility, readability, layout and response format of the tool. The guideline was pilot-tested on the first participants to see if any changes were needed, which turned out not to be necessary.
Why did you choose the analysis by Graneheim and Lundman? Please provide context/reasoning here. Also, which software did you use? Where the interviews transcribed?	We chose Graneheim and Lundman's approach since it is frequently cited in studies using content analysis. We have also used this method in previous studies and found it very helpful. We didn't use any software. We have added that the first author (NE) transcribed all the interviews to the manuscript.<br><br>NE transcribed the interviews and read through the transcribed interview texts several times to obtain a sense of the whole.
I am missing a socio demographic overview of the participants: how many were female, what age groups, etc. Please provide that context at the beginning of the result section.	Thank you for making us aware of this. We have now added a table with more information on the background of the participants.<br><br>Table 1. Gender and role of interviewees

Table 1. Gender and role of interviewees

N=11	**Gender**	**Representing**
	Female	Patient representa
	Male	Patient representa
	Female	Patient representa
	Female	Patient representa

<table>
<tr><td></td><td>
<table>
<tr><td></td><td>Female</td><td>RN A</td><td></td></tr>
<tr><td></td><td>Female</td><td>RN B</td><td></td></tr>
<tr><td></td><td>Female</td><td>RN C</td><td></td></tr>
<tr><td></td><td>Female</td><td>RN D</td><td></td></tr>
<tr><td></td><td>Female</td><td>RN E</td><td></td></tr>
<tr><td></td><td>Female</td><td>Physician A</td><td></td></tr>
<tr><td></td><td>Male</td><td>Physician B</td><td></td></tr>
</table>
</td></tr>
<tr><td>Also, you jump right into the categories and are quickly deep into the results itself. I always prefer a bit of context here, how long were the interviews, how did the subcategories evolve etc. Please provide a bit more “background” information. If I have that information I am able to go into the results in more detail and check again content wise and if clear to me.</td><td>Thank you for raising this question. We tried to be as detailed as possible in the procedure section and think that the information you ask for actually is described:
Length of interviews (under the heading procedures): “The interviews were terminated when the interviewer judged that no new information could be gleaned or at the request of the interviewee. All sessions were digitally recorded and lasted 40 – 78 minutes (mean 58 min)”.

Evolvement of subcategories: ”Subsequently, meaning units comprising words, sentences or phrases corresponding to the defined main categories were identified. In the next step, meaning units were condensed, coded and sorted into sub-categories for each of the main categories. To improve the trustworthiness of the analyses, at least two of the authors (CT, AF) collaborated with the first author (NE) regularly in the analysis and discussed their results until consensus was obtained”.</td></tr>
<tr><td>I am missing a kind of point what type of changes you are going to do now after this testing and what this entails for the new updated version you want to test psychometrically. Please provide more in depth details here, a kind of implication section.</td><td>Some changes we made in the instrument are described in detail in table 3, and also mentioned in the discussion, as follows:
To discriminate between good care and person-centred care it was suggested that the tool should place greater emphasis on eliciting and addressing both patient’s capabilities and resources as well as their psychosocial needs to better reflect their importance in the gPCC framework. Therefore, the activity, resources and capability parts in the instrument were expanded, please see table2.
A defining feature in the framework is to build a partnership, which was also</td></tr>
</table>

	suggested to be more prominent in the instrument, both by promoting patients´ self-efficacy by identifying and utilizing their personal capabilities, as well as by supporting their psychosocial needs<br>• Some of the participants found it difficult to understand the ”doesn´t do” alternative, intended for designating activities that did not appear in the interactions, and so we have clarified its use in the instructions<br>• Some words such as ”paraverbal” and “mutual gaze” were not understood by all participants and were therefore replaced with more common words in line with recommendations<br>• The participants expressed a slight preference for a 5-point Likert scale with a neutral midpoint to our 4-point scale
- Please provide the English translation for the Swedish reference #23	We have now provided the English translation for the Swedish reference #23<br><br>The National Board of Health and Welfare. A more accessible and patient-centered care (2016). Summary and analysis of the county councils' and regions' action plans - interim report. Swedish National Board of Health and Welfare
**Reviewer #2:**	**Remarks:**
Well done, another strong body of work coming from Sweden. I do enjoy reviewing this work. Overall, a strong article.	Thanks for the kind words!
Please change to article rather than paper	We have now changed to article instead of paper.<br><br>This article presented a newly developed direct observation tool for this purpose and evaluated its content and usability for assessing competency in the delivery of person-centred care.
I am a little concerned about the recruitment method. Using a network from one researcher is not an overly rigorous method or approach. I wonder if you might better explain this as a snowball sample or other more academic language?	Thanks for pointing this out. We’ve revised the text as follows: “The eligible participants were recruited from February 2022 to April 2022 through a combination of network and snowball sampling.
Interesting use of technology and think aloud- strong work here	Thank you very much for this nice comment!

What you have done here is a review of person-centred practice. I know the literature tends to amalgamate PCC and PCP as one and the same, but I see them as two separate (and equally important) elements. Person-centred care is the structures and environment around which centredness can exist (you alluded to accreditation etc in the background). PCP on the other hand is the practice elements that professionals bring. Please consider how separating these terms, or at least giving some nod to the difference in this paper. I have written on this topic myself but is still under review (I appreciate that is of little help to you now), but something I think needs to be considered in this space moving forward.	Thank you for raising this issue, we have now added a sentence in the discussion.<br>One of the core aspects in person-centered practice has been described by McCormack as "being in relation" and "being in place" which emphasizes the importance of relationships and to be interconnected with ones´ social world (48).<br><br>We agree and have added this footnote at page 6: [1] Although differences exist in the definitions of person-centred care and patient-centred care, the terms are frequently used interchangeably in the literature. For the sake of parsimony, the term person-centred care has systematically been used in this article.
**Reviewer #3:**	**Remarks:**
Thanks for giving me the opportunity to read and comment on this interesting paper. This small-scale qualitative study set out evaluate the content and usability of a new direct observation tool for assessing competency in delivering person-centred care based on the Gothenburg Centre for Person-Centred Care framework, by using think-aloud techniques with 11 informants, both patients and professionals. The manuscript is topical and should be interesting and relevant for researchers, clinicians and educators. A strength of the study is the integration of PCC theories and clinical work. The manuscript fits well into the aims and scope of BMJ Open. It has a good structure, is well written and easy to read and follow.	Thank you so much for the kind words!
The title and key words are well chosen, and the abstract summarizes the manuscript in a good way.	Thank you for this comment!
The introduction and literature-reviews are well written, cover several important points and provides a good overview of the area. The authors use a number of references referring to	We agree that the concepts can be more clearly described. We have added a footnote explaining our focus on person-centred care at page 6: [1]

patient-centred care, a concept which indeed has many similarities with person-centre care, but also differences. Please see if some of these references could be exchanged to fit better with the person-centred approach of the manuscript.	Although differences exist in the definitions of person-centred care and patient-centred care, the terms are frequently used interchangeably in the literature. For the sake of parsimony, the term person-centred care has systematically been used in this article.<br>We have now deleted the abbreviation PCC and replaced with person-centred care throughout the manuscript.
The study rationale is clearly described.	Thank you for this nice remark.
Why were this number of informants chosen? Do you consider data to be saturated (or if you dislike this GT-concept, you could use "information power" a concept launched by Malterud et al 2016)?	Although we cannot make any claims of reaching saturation in any of these categories, the richness of the data suggests we came a long way and that the number of participants were chosen was adequate. We felt that saturation had been approached in the data because later probing interviews added little new information to what earlier probing interviews had already provided. We have now commented on this in the limitations section.<br><br>Another limitation is that we cannot make any claims of reaching saturation in any of these categories, the richness of the data suggests on the other hand that we came a long way and that the number of participants chosen was adequate. We judged that saturation had been approached in the data because later probing interviews added little new information to what earlier probing interviews had already provided.
I would like some more information about the participants, like age, gender, years in the profession (if applicable) etc. This demographic information could be displayed in a table.	Thank you for making us aware of this. We have now added a table with a socio demographic overview of the participants.
Why use convenience sampling?	Thank you for noticing this. We have changed it to purposeful sampling.<br><br>The eleven participants were recruited in western Sweden and the inclusion criteria was that participants with experience of receiving, working with and/or implementing person-centred care were recruited using purposeful sampling and selected to represent a variety of stakeholders and potential end-users.

The description of the data analysis should be more comprehensive, to enhance trustworthiness. The steps in the analysis could be displayed in a table or figure, with examples of coding and categorisation.	We find it difficult to present the analysis in a table or figure and think it is presented quite in detail in the procedure and analysis. We have, however, expanded both of these paragraphs with a few sentences.<br>NE both transcribed the interviews and read through the transcribed interview texts several times to obtain a sense of the whole.<br>No coding software was used.
The finding are well described, and the section is easy to read and follow. The quotes are illustrative. I would however have liked if you differentiated between the informants, like RN A, Patient B etc. This to enhance confirmability, that the findings are based on contributions from all informants and not just a few.	Thank you for raising this issue, we have now changed in line with your suggestion (added a new table (Table 1), and in relation to the quotes). The table has also been added above in response to another comment.
The discussion is well written and interesting.	Thank you.
The study strength and weaknesses should be addressed. Preferably in relation to criteria for quality assessments of qualitative studies. You could use the CORTEQ guidelines or Lincoln & Guba's quality criteria for qualitative studies. One limitation is the use of convenience sampling. A strength is the inclusion of both patients and professionals.	Thank you for this remark, we apologize for not including the COREQ checklist. We have stated in the strength and weakness section that "A strength in the present direct observation tool is that patients represented almost half of the participants in both development studies. Although patient involvement in the development of person-centred care assessment instruments has long been emphasized (54), none of the existing person-centred care direct observation tools were developed with patients or their families".<br><br>But we have now added the limitations "Another limitation is that we cannot make any claims of reaching saturation in any of these categories, the richness of the data suggests on the other hand that we came a long way and that the number of participants chosen was adequate. We assessed that saturation had been approached in the data because later probing interviews added little new information to what earlier probing interviews had already provided".

VERSION 2 – REVIEW

**REVIEWER**	Byrne, Amy-Louise<br>Central Queensland University School of Nursing and Midwifery
**REVIEW RETURNED**	23-May-2024

**GENERAL COMMENTS**	Thank you

**REVIEWER**	Holmstrom, Inger<br>Maladalen University Sweden, School of Health, Care and Social Welfare
**REVIEW RETURNED**	20-May-2024

**GENERAL COMMENTS**	The authors have attended to the other reviewers' and my comments in a constructive way, and the manuscript has improved. I have no further comments or queries.

VERSION 2 – AUTHOR RESPONSE
